# Metamaterial Behavior of Polymer Nanocomposites Based on Polypropylene/Multi-Walled Carbon Nanotubes Fabricated by Means of Ultrasound-Assisted Extrusion

**DOI:** 10.3390/ma9110923

**Published:** 2016-11-14

**Authors:** Juan C. Pérez-Medina, Miguel A. Waldo-Mendoza, Víctor J. Cruz-Delgado, Zoe V. Quiñones-Jurado, Pablo González-Morones, Ronald F. Ziolo, Juan G. Martínez-Colunga, Florentino Soriano-Corral, Carlos A. Avila-Orta

**Affiliations:** 1 Innovación y Desarrollo en Materiales Avanzados A. C., Grupo POLYnnova, Carr. San Luis Potosí-Guadalajara 1510, Nivel 3, Local 12, Lomas del Tecnológico, San Luis Potosí, S.L.P. C.P. 78211, Mexico; jcarlospm40@hotmail.com (J.C.P.-M.); miguel.waldo@polynnova.mx (M.A.W.-M.); zoe.vineth@polynnova.mx (Z.V.O.-J.); 2 Centro de Investigación en Química Aplicada, Blvd. Ing. Enrique Reyna H. 140, Col. San José de los Cerritos, Saltillo, Coahuila C.P. 25294, Mexico; victor.cruz@ciqa.edu.mx (V.J.C.-D.); pablo.gonzalez@ciqa.edu.mx (P.G.-M.); rziolo@cs.com (R.F.Z.); guillermo.martinez@ciqa.edu.mx (J.G.M.-C); florentino.soriano@ciqa.edu.mx (F.S.-C.)

**Keywords:** polypropylene, carbon nanotubes, melt flow index, extrusion, ultrasound, AC electrical properties, negative dielectric constant, metamaterials

## Abstract

Metamaterial behavior of polymer nanocomposites (NCs) based on isotactic polypropylene (iPP) and multi-walled carbon nanotubes (MWCNTs) was investigated based on the observation of a negative dielectric constant (ε′). It is demonstrated that as the dielectric constant switches from negative to positive, the plasma frequency (ω_p_) depends strongly on the ultrasound-assisted fabrication method, as well as on the melt flow index of the iPP. NCs were fabricated using ultrasound-assisted extrusion methods with 10 wt % loadings of MWCNTs in iPPs with different melt flow indices (MFI). AC electrical conductivity (σ_(AC)_) as a function of frequency was determined to complement the electrical classification of the NCs, which were previously designated as insulating (I), static-dissipative (SD), and conductive (C) materials. It was found that the SD and C materials can also be classified as metamaterials (M). This type of behavior emerges from the negative dielectric constant observed at low frequencies although, at certain frequencies, the dielectric constant becomes positive. Our method of fabrication allows for the preparation of metamaterials with tunable ω_p_. iPP pure samples show only positive dielectric constants. Electrical conductivity increases in all cases with the addition of MWCNTs with the largest increases observed for samples with the highest MFI. A relationship between MFI and the fabrication method, with respect to electrical properties, is reported.

## 1. Introduction

Isotactic polypropylene (iPP) is a nonpolar polyolefin with high electrical resistivity and used in the fabrication of energy storage materials [1]. Its chemical structure is composed by a backbone chain with methyl groups located on only one side of the chain.

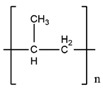
(1)

Particularly, metalized biaxially-oriented polypropylene films are widely used in the capacitor industry due its high breakdown voltage, although its dielectric constant or permittivity (ε′) is about 2. This behavior changes dramatically when polypropylene is mixed with nanoparticles, particularly with multi-walled carbon nanotubes (MWCNTs), which usually increase the value of ε′. For example, ε′ increases up to 600 during annealing at high temperature and drastically diminishes from 700 to 92 during crystallization from the molten state [2], a similar trend is seen in the electrical conductivity (σ) when 2 wt % of MWCNT are used. Logakis et al. [3] found a value close to 20 for samples containing 1.5 wt % of MWCNTs. On the other hand, it has been determined that upon increasing the amount of MWCNTs in blends with iPP, ε′ increases, showing values up to 100 for samples containing 5 wt % of nanotubes [4], while in another study, [5] a value of 14 was found for samples with 2 wt %. Higher ε′ values have also been found for blends of iPP/HDPE/MWCNTs, such as 10^3^ for the blend 70/30/1 [6]. Likewise, iPP/reduced graphene oxide showed a dielectric constant in the order of 10^3^ [7].

In all of the cases cited above, positive values of the dielectric constant were observed. However, unexpected behavior was reported in recent years where negative values of ε′ were obtained for polymer nanocomposites (NCs) based on carbon nanoparticles, including MWCNTs. NCs show a negative dielectric constant at low frequencies and a positive dielectric constant at high frequencies. Such behavior is characteristic of materials called metamaterials since they show properties that cannot be found in natural materials [8]. Metamaterials origins can be traced back to mid-late 1960s with the pioneering theoretical approach done by Veselago [9,10] of materials having simultaneous negative values of the dielectric and magnetic susceptibilities, and then followed by theoretical and experimental work done by Pendry’s group in the 1990s [11,12]. Additionally, it has been reported that carbon nanotube mats exhibit negative values of the dielectric constant [13]. A similar behavior has been shown for conductive polymer/carbon nanotubes nanocomposites [14,15,16,17,18] and for iPP/carbon nanotube nanocomposites [19]. However, in most cases, polymer/carbon nanotube nanocomposites were prepared by solution methods, where it is expected that MWCNTs are fully dispersed. Other preparation methods, such as the ones based on melt extrusion, typically render a poorer dispersion of carbon nanotubes compared to the one obtained by solution methods, with the consequence of the need to use larger amounts of nanoparticles to obtain acceptable or comparable properties. In this sense, Ramasamy [20] prepared polypropylene/graphene nanocomposites and observed negative values of the dielectric constant using amounts of 30%–40% of graphene. It was recently shown that the dispersion of nanoparticles in polymer matrices can be highly enhanced using ultrasound-assisted methods [21,22], thus opening the possibility of reducing the amount of nanoparticles to obtain metamaterial behavior.

In polymers containing graphene, reduced graphene oxide and carbon nanotubes a percolation effect drastically reduces the resistivity of polymers by several orders of magnitude. To explain this effect, it has been suggested that a critical amount of conductive fillers, such as those of graphene, allow an increase in dielectric constant due to the polarization effect at the interface of polypropylene and graphene particles. However, in amounts as high as 35 wt %, which allow the percolation effect, the charge accumulation at the interface of polypropylene and graphene results in a negative dielectric constant at low frequencies [20]. Based on the negative dielectric constant of metals, Zhang et al. [19] suggested a similar behavior in the case of iPP/MWCNTs NCs by studying the relationship between negative permittivity and the plasma frequency ω_p_. Two plasma frequencies were reported in the case of polypyrrole, which were attributed individually to the most delocalized electrons and to confined electrons [23]. It was the negative dielectric constant observed below the first plasma frequency that was attributed to the plasma-like resonance of free electrons. In contrast, polypropylene/reduced graphene oxide was prepared with a percolation threshold of 0.03 vol % using a latex method, and then with higher amounts of fillers, and did not show a negative dielectric constant; rather, a drastic increase with frequency was observed [7].

In a previous study, we reported the morphology and DC electrical resistance of iPP and of iPP/MWCNT high-loading nanocomposites [22]. We demonstrated that the melt flow index of iPP and the ultrasound-assisted extrusion method were key variables in producing iPP/MWCNT NCs that could be classified as insulators, static-dissipative, or conductive materials. A further investigation of the electrical behavior of the above-classified NCs as a function of frequency was conducted in the present study.

## 2. Results

### 2.1. Electrical Properties of iPP

**Figure 1:**
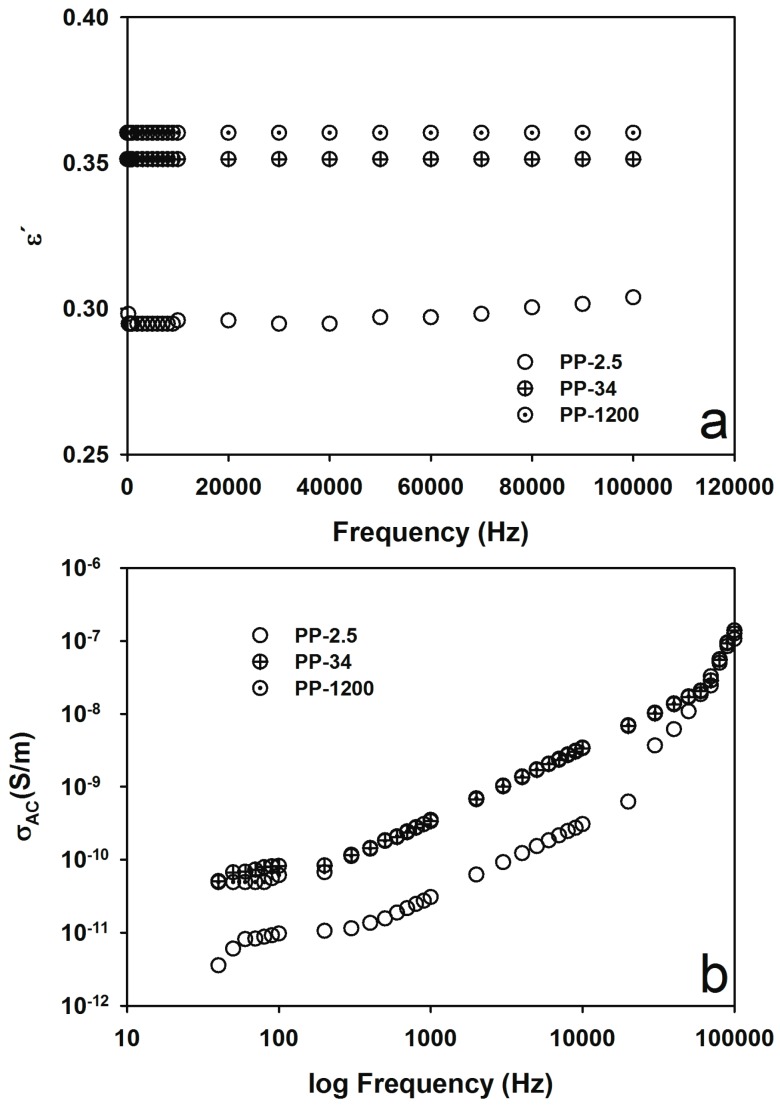
Electrical properties of iPP with different MFIs (2.5, 34, and 1200 g/10 min). (**a**) ε′ and (**b**) σ_(AC)_.

Electrical properties of iPP with different MFIs were evaluated prior to the NC’s fabrication. The dielectric constant (ε′) and the electrical conductivity (σ_AC_) are shown in Figure 1. In Figure 1a, ε′ values for the three different polymers with different MFIs are close to each other (within 0.3 and 0.4) and almost constant through the scanned frequencies. These values are lower than the 2.2 values usually reported in the literature for iPP and probably due to the sample preparation. On the other hand, the conductivity, σ_AC_, increases from ca. 10^−11^ S/m at low frequencies to 10^−9^ S/m at high frequencies, as seen in Figure 1b. The values obtained for the three polymers correspond well to the behavior of electrical insulators [3,24,25] with low dielectric constant.

### 2.2. Elecrical Properties of iPP/MWCNT Polymer Nanocomposites

Although only the ε′ and electrical conductivity σ_(AC)_ will be discussed for the iPP/MWCNTs NCs fabricated using three different iPPs with different MFI and four ultrasound-assist extrusion methods, other properties were obtained and are reported in the Supplementary Materials; these include dielectric loss (ε′′), tan δ, real and imaginary impedance (Z′ and Z′′), and electrical modulus (M′ and M).

#### 2.2.1. Dielectric Constant, ε′

**Figure 2:**
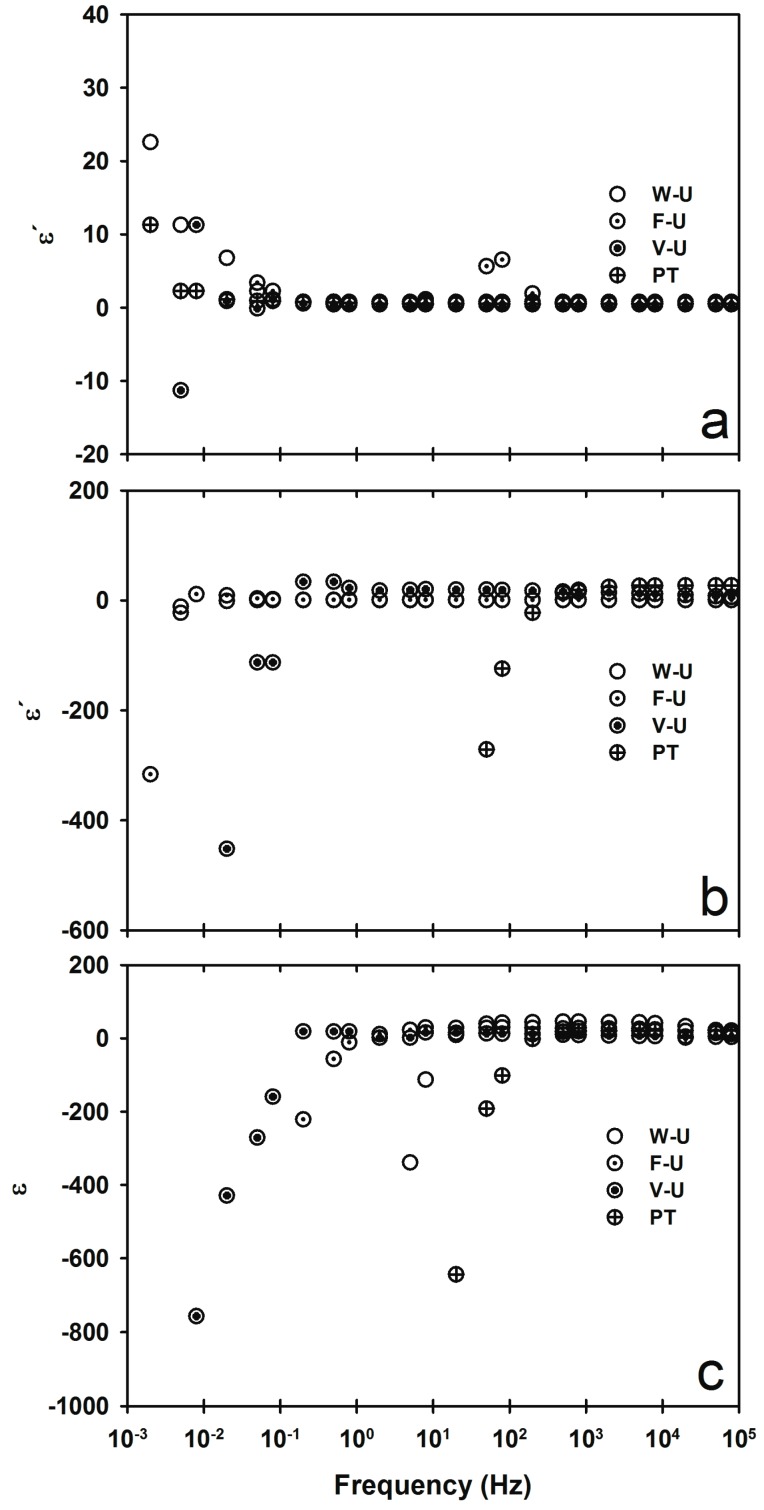
ε′ for (**a**) iPP_MFI=2.5_/MWCNT; (**b**) iPP_MFI=34_/MWCNT; and (**c**) iPP_MFI=1200_/MWCNT fabricated using different ultrasound-assisted extrusion methods.

The dielectric constant for iPP/MWNT polymer nanocomposites fabricated with different ultrasound-assisted methods is shown in Figure 2 (a) iPP_MFI=2.5_; (b) iPP_MFI=34_; and (c) iPP_MFI=1200_. In the case of the sample with the lowest MFI (Figure 2a), ε′ shows no significant change in behavior as compared with the pure polymers with values range from 0.7 to −10. On the contrary, in the case of the polymer nanocomposites where the highest MFI was used, iPP_MFI=1200_, a drastic change of behavior is observed (Figure 2b). All samples, regardless of the fabrication method, show a negative dielectric constant (see Table 1) in some cases as low as 10^7^. However, the change from negative to positive values, ω_p_, is highly dependent on the ultrasound-assisted fabrication method, as shown in Table 1. In the case of the samples fabricated using intermediate iPP_MFI=34_, the ones fabricated using V-U and PT show a similar behavior compared to the ones of iPP_MFI=1200_, while the one fabricated without ultrasound (W-U) behaves similarly to that of iPP_MFI=2.5_.

In view of the above results, we note that MFI and the ultrasound-assisted melt extrusion method play key roles in obtaining materials with negative dielectric constants and on the plasma frequency value of the material. Insulator-like materials obtained using a low MFI iPP (2.5 g/10 min) do not show negative ε′ or the values are quite low as compared with the static dissipative and conductive materials prepared with higher MFI (34 and 1200 g/10 min). Moreover, we note that the plasma frequency obtained for the static-dissipative materials is orders of magnitude lower than that observed for the conductive materials, suggesting that the former are capable of electrical charge storage at low frequencies.

#### 2.2.2. Electrical Conductivity, σ_(AC)_

**Figure 3:**
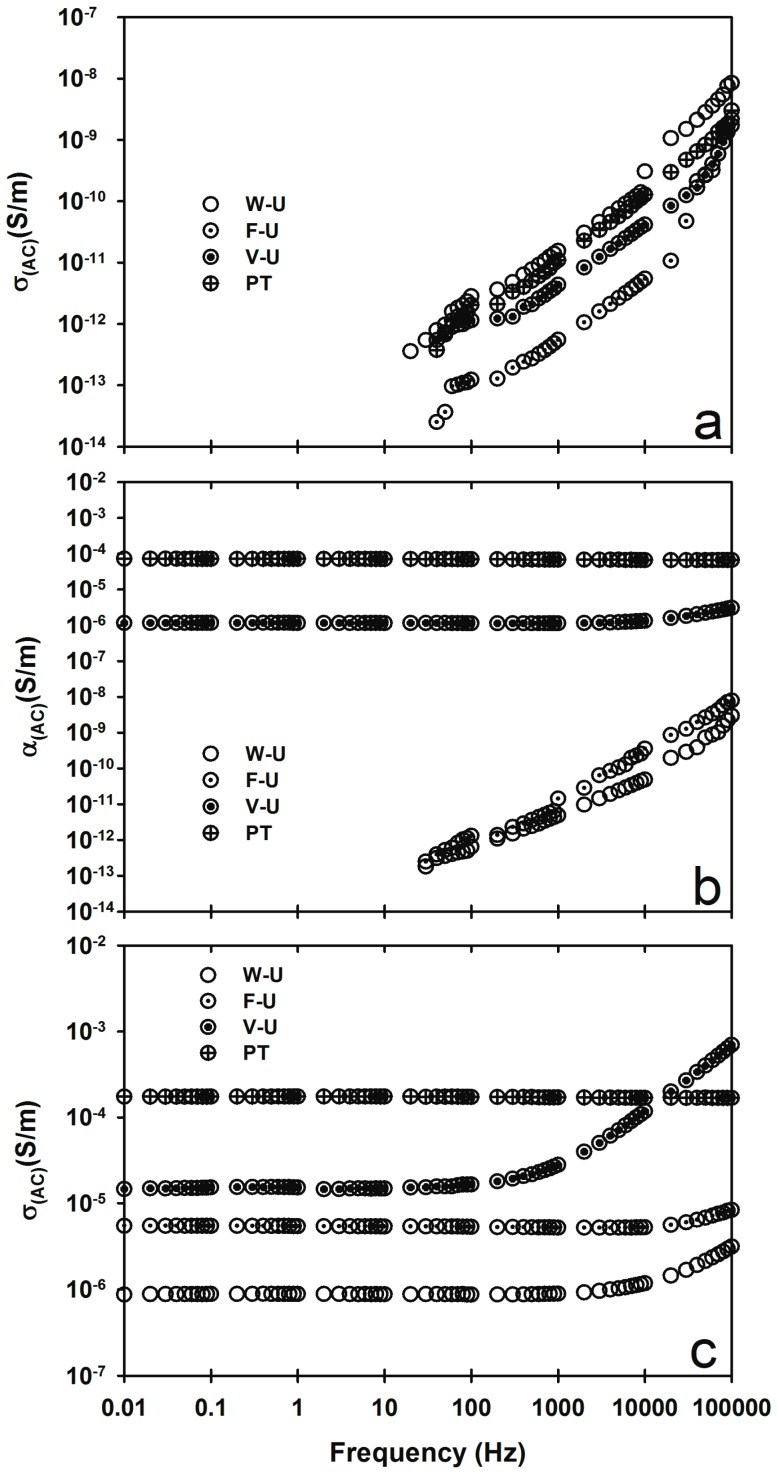
σ_(AC)_ for (**a**) iPP_MFI=2.5_/MWCNT; (**b**) iPP_MFI=34_/MWCNT; and (**c**) iPP_MFI=1200_/MWCNT fabricated using different ultrasound-assisted extrusion methods.

Electrical conductivity σ_(AC)_ was obtained in the sweep range of frequencies from 0.002–100,000 Hz and the results are shown in Figure 3. In Figure 3a, we note that the samples fabricated with iPP_MFI=2.5_ show a similar insulator-like behavior like that of pure iPP, regardless of the fabrication method. Interestingly, the samples fabricated using iPP_MFI=34_ and V-U and PT fabrication methods (Figure 3b) show an almost constant behavior through the frequency range typical of conductive materials with σ_(AC)_ values close to 10^−6^ and 10^−4^ S/m, respectively, while the samples fabricated with W-U and F-U methods show an insulator-like behavior similar to the one shown for the pure iPPs and for samples fabricated using iPP_MFI=2.5_. In contrast, regardless of the fabrication method, the samples fabricated using iPP_MFI=1200_ show a near constant behavior with a slight increase at high frequencies with σ_(AC)_ values of 10^−4^, 10^−5^, 10^−5^, and 10^−6^ for samples fabricated using PT, V-U, F-U, and W-U methods, respectively. Nanocomposites of iPP/graphene show σ_(AC)_ ca. 10^−4^ S/cm [20], using 30 wt % or higher of graphene. However, the fabrication of iPP/MWCNT with 10 wt % of fillers is important because a high loading of filler can reduce the mechanical properties of PP/CNT [22]. It can be concluded that the fabrication method plays a significantly important role in the AC electrical conductivity, σ_(AC)_, of the material.

#### 2.2.3. Voltage Effect on ε′ and σ_(AC)_

**Figure 4:**
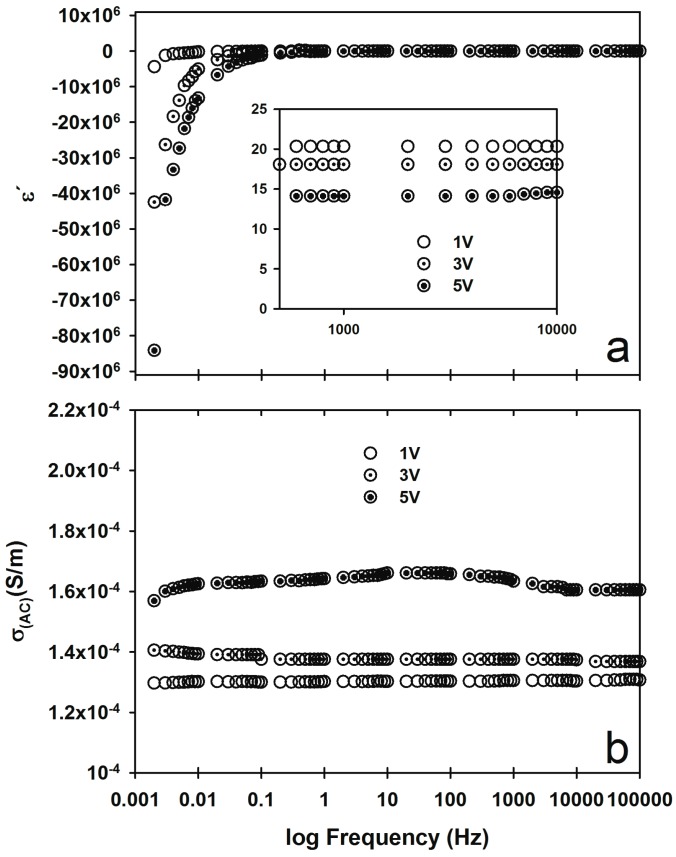
Electrical properties of iPP_MFI=1200_/MWCNT obtained by the PT fabrication method, (**a**) ε′ and (**b**) σ_(AC)_.

The sample iPP_MFI=1200_/MWCNT fabricated with the PT method was subjected to different test conditions to determine their effect on the electrical properties. Electrical properties were evaluated using 1, 3, and 5 V peak-to-peak AC. The dielectric constant and electrical conductivity results are shown in Figure 4. In the inset of Figure 4a, it can be seen that ε′ decreases when the applied test voltage increases from 20.3 at 1 V to 14.1 at 5 V. Additionally, the switch from positive to negative ε′ frequency (ω_p_) increases with low voltages evaluated from 30 Hz with 1 V to 500 Hz with 5 V. The AC conductivity (Figure 4b) increases by 0.00003 S/m when the applied voltage is increased from 1–5 V. Typically, 1 V is used for electrical evaluation of capacitors. In the present work, however, our goal is to enhance the differences observed for the electrical behavior of high-resistance samples, so the evaluation voltage was fixed to 5 V for all of the analyses. The effect of voltage on the electrical properties indicates that is possible to control the electrical properties with the applied voltage. For example, to change from negative to positive ε′ at the same frequency it is necessary only to increase or decrease the applied voltage.

## 3. Discussion

The results demonstrate a metamaterial behavior for iPP/MWCNT polymer nanocomposites at a fixed MWCNT content of 10 wt % in both the static-dissipative and conductive phases. The behavior is more evident in the conductive materials with the larger negative values of ε′, as well as the shift to higher frequencies for ω_p_. The type of electrical behavior is also dependent, in turn, on the MFI of the iPP and on the fabrication method. In a previous report [20], the dielectric constant behavior was studied as a function of the graphene content at different frequencies. It was shown that an amount of 30 wt % or 40 wt % of graphene was needed to obtain a negative dielectric constant and the crossover from negative to positive was ca. 200 Hz., at 1 V. It has also been reported that gelled PP with xylene at 2 wt % CNT present negative ε′ values [19]. In the present study, a similar behavior was obtained with 10 wt % of MWCNTs, suggesting that the negative ε′ behavior is driven by the dispersion of MWCNTs in the polymer matrix and by applied voltage, rather than by the filler amount. 

It is well know that metamaterials comprise assemblies of composite materials where a precise assembly or arrangement is required to manipulate electromagnetic waves. The question arises on the type of assembly or arrangement that can lead to such behavior in the metamaterials. Aiming to further investigate the dispersion of MWCNTs in iPP, SEM micrographs were taken at different magnifications on selected samples where the MFI = 34 g/10 min. The reason for this choice is based on the fact that this set of samples shows insulating, static-dissipative, and conductive behavior, as well as metamaterial behavior in the last two cases. Due to the moderate content of MWCNT in the samples, agglomerates are expected and have been observed [22]. 

**Figure 5:**
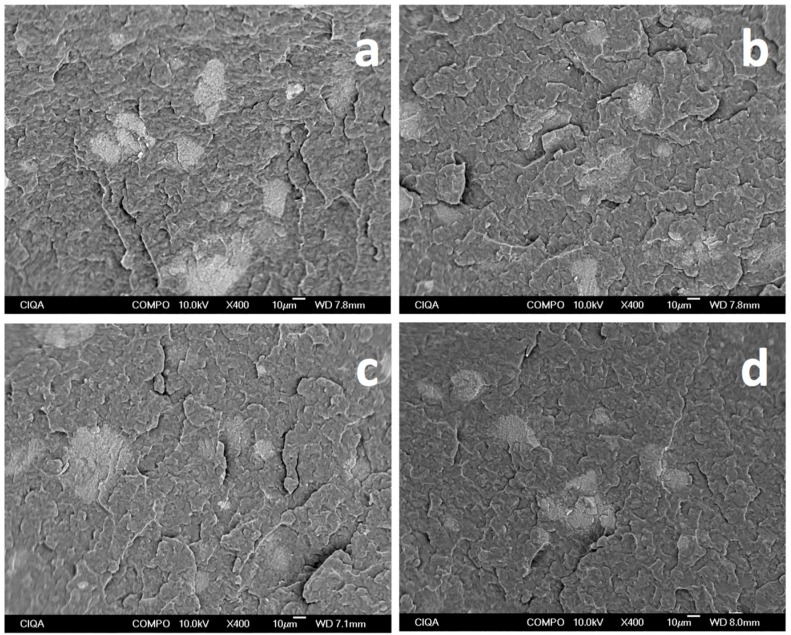
Low-magnification SEM micrographs for iPP_MFI=34_/MWCNT fabricated using different ultrasound-assisted extrusion methods (**a**) W-U; (**b**) F-U’; (**c**) V-U; and (**d**) PT.

For a first approximation, overall images of samples were taken at low magnifications as shown in Figure 5. Agglomerates can be observed as white spots larger than 10 μm, where the largest ones are observed for the sample without ultrasound-assisted W-U, and the smallest, in the case of PT method, with intermediate-sized agglomerates for the case of F-U and V-U. A broad correlation can be made that the smaller the agglomerates, the higher the electrical conductivity and negative permittivity.

**Figure 6:**
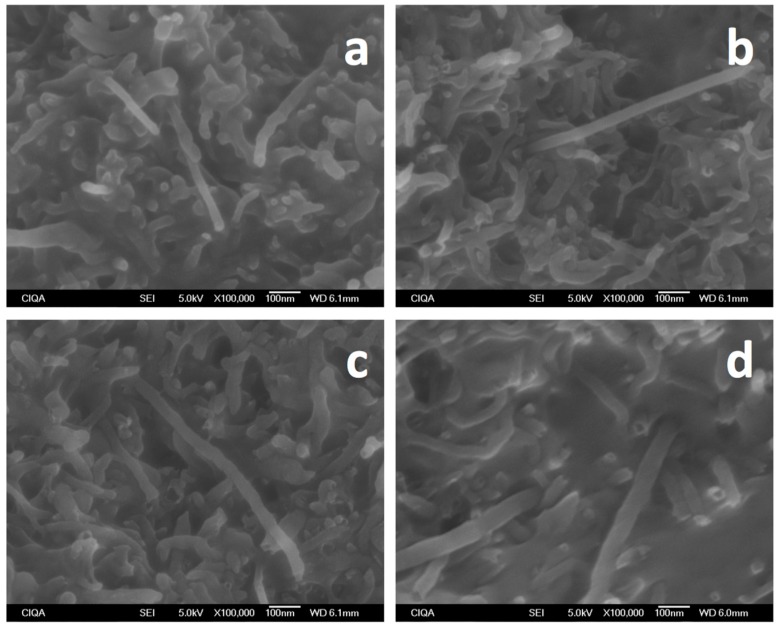
SEM micrographs within agglomerates for iPP_MFI=34_/MWCNT fabricated using different ultrasound-assisted extrusion methods (**a**) W-U; (**b**) F-U; (**c**) V-U; and (**d**) PT.

**Figure 7:**
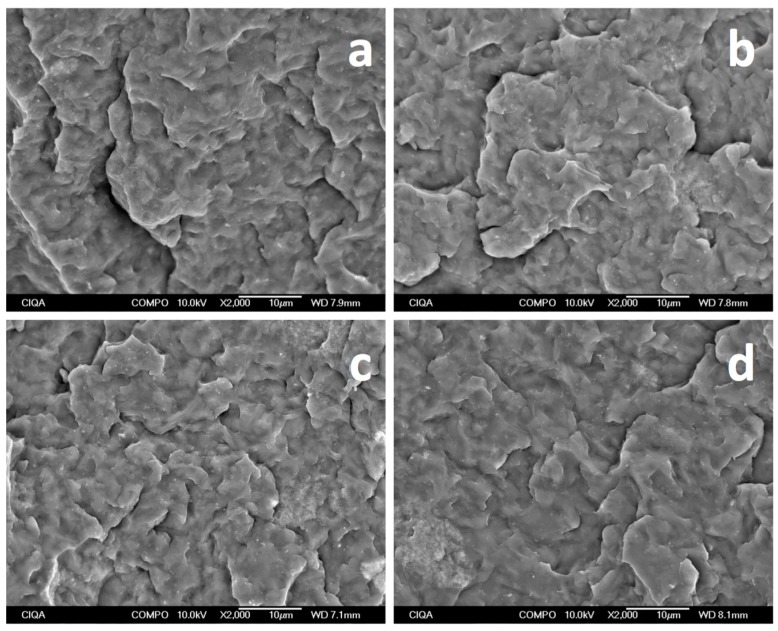
SEM micrographs taken between agglomerates for iPP_MFI = 34_/MWCNT fabricated using different ultrasound-assisted extrusion methods (**a**) W-U; (**b**) F-U; (**c**) V-U; and (**d**) PT.

A closer view within agglomerates (Figure 6) suggests that individual MWCNTs are separated more from each other and some of them extend from the bundles, as in the case of conductive (PT) and static-dissipative (F-U and V-U) samples, more so than in the insulating samples (W-U). Moreover, in a recent study for iPP/MWCNTs fabricated using the solution mixing method [26], the idea was proposed of nanotube wrapping by iPP in samples with low molecular weight and a moderate loading of nanotubes (2–4 wt %) showing a fibrillar morphology due to a match of the helical structure of both components. In that study, we note that agglomerates were not observed due to the fabrication method. Therefore, it might be possible that wrapping might be a possibility in samples where MWCNTs are separated from each other within the agglomerates, forming a specific assembly as investigated for the case of polyesters [27], which can lead to a negative dielectric constant. In fact, significant interaction of iPP and MWCNTs can be observed in Figure 6d. Zones between agglomerates were also observed (Figure 7) aiming to determine whether or not pathways of MWCNTs exist between them that can contribute to a percolation network. Clear evidence of differences between the samples, however, could not be found, although it is expected that individual nanotubes or small bundles exerted from large agglomerates due to the ultrasound action are present in these zones, mostly for static-dissipative and conductive samples than for insulating ones.

To conclude, we believe that the use of ultrasound renders low-density agglomerate samples with exerted individual nanotubes from agglomerates, which, in turn, produce static-dissipative and conductive samples with a negative dielectric constant. Moreover, our method of fabrication allows for the preparation of metamaterials with tunable ω_p_. The negative ε′ is an essential key to creating metamaterials with a negative refractive index or artificial negative index materials (NIMs). Such materials have generated considerable attention because of their unique electromagnetic properties [28,29,30]. Further studies on melt- and solution-fabricated materials using different concentrations of MWCNTs are recommended to further understand possible electromagnetic conduction pathways in the materials and to fabricate additional materials with tunable plasma frequencies.

These types of polymer nanocomposites fabricated with a high melt flow index have potential applications in textile fibers and, for those with negative dielectric constant, for potential use in extremely low frequency (ELF), low-loss communication applications.

## 4. Materials and Methods

Materials: Three iPP homopolymers with different MFI were used. The homopolymers were provided by: Exxon, (Houston, TX, USA) grade PP4712 E1 (MFI = 2.5 g/10 min), Indelpro, (Altamira, Mexico), grade PL835N (MFI = 34 g/10 min), and Indelpro (Altamira, Mexico), grade Profax PL 505 (MFI = 1200 g/10 min). The three materials were designated as iPP_MFI=2.5_, iPP_MFI=34_, and iPP_MFI=1200_, respectively, according to their manufacturer-reported MFI. Multi-walled carbon nanotube-grade IGCNTs were purchased from Cheaptubes, Cambridgeport, VT, USA, with an outer diameter of 20–40 nm with a length of 10–30 μm and 90% purity.

Methods: iPP/MWCNT nanocomposites with 10 wt % of MWCNT were fabricated using a laboratory twin-screw extruder, Thermo Scientific model 24MC (Stone, UK) with an L/D = 40:1, a speed of 100 RPM, and a plain temperature profile of 220 °C along the barrel. Four different fabrication methods were used to fabricate the polymer nanocomposites. The first one was carried out without ultrasound and designated as W-U. In the second and third methods, an ultrasound probe was attached to the extruder and a fixed frequency (F-U) and variable frequency (V-U) was applied to the molten materials in the second and third methods, respectively. In the fourth method, MWCNTs were treated in a fluidized airbed with an ultrasound probe before being used in the fabrication of the nanocomposites and was designated as PT for pretreatment. Further details of the preparation methods can be found elsewhere [22,31,32].

Characterization techniques: AC electrical measurements: Electrical measurements of masterbatch plates were evaluated in an LCR (inductance (L), capacitance (C) and resistance (R)) meter Keysight (Model E 4980 A, Santa Rosa, CA, USA) over 20 Hz to 2 MHz and LCR meter Model ZM2372, from 0.001 Hz to 100 kHz. The samples were cut into 1 cm^2^ squares and both sides were covered with silver conductive paint to form an electrode. The measurements were made at room temperature; Scanning Electron Microscopy (SEM): Small pieces of compression-molded plaques were cryo-fractured and coated with gold. SEM micrographs were taken directly of the cryo-fractured surface of the iPP/MNT nanocomposites using a field emission scanning electron microscope, JSM-74101F-JEOL VR (JEOL, Tokyo, Japan) with a secondary electron detector (SEI) using a voltage of 4.0 kV; Transmission Electron Microscopy (TEM): TEM micrographs were obtained using a TEM Titan 89–300 (FEI, Hillsboro, OR, USA) of MWCNTs previously dispersed in acetone under ultrasonication for 15 min.

## 5. Conclusions

The present study showed that methods used for the ultrasound-assist extrusion fabrication influence the electrical properties of isotactic polypropylenes (iPP) with different MFI and of nanocomposites (NCs) with 10 wt % loadings of multi-walled carbon nanotubes using the same polymers. It was demonstrated that the selection of the melt flow index of PP, coupled with an ultrasound-assisted extrusion method, can render metamaterial behavior. Pure polymers presented an insulator-like behavior, while it was concluded that the fabrication method, as well as the iPP melt flow index, play key roles in determining the metamaterial behavior of NCs. Ultrasound-assist melt extrusion methods appear to enhance the dispersion of MWCNTs and produce low MWCNTs density agglomerates, which, in turn appear to enhance the static-dissipative and conductive behavior of the NCs. Negative dielectric constants are observed, which is indicative of metamaterial behavior, with the fabrication method allowing for a tunable plasma frequency. The same trend was found increasing the melt flow index of polypropylene.

## Figures and Tables

**Table 1:** Reading from top to bottom: type of electrical material ^1^, crossover frequency from negative to positive (ω_p_), negative ε′ maximum value, and metamaterial classification for iPP/MWCNT nanocomposites fabricated by different ultrasound-assisted methods.

Sample	Fabrication Method			
	W-U	F-U	V-U	PT
iPP_MFI=2.5_/MWCNT	Insulator	Insulator	Insulator	Insulator
	N/A	N/A	N/A	N/A
	N/A	N/A	N/A	N/A
	N/A	N/A	N/A	N/A
iPP_MFI=34_/MWCNT	Insulator	Static-dissipative	Static-dissipative	Conductor
	N/A	0.006 Hz	0.1 Hz	400 Hz
	N/A	−10^2^	−10^3^	−10^7^
	N/A	Metamaterial	Metamaterial	Metamaterial
iPP_MFI=1200_/MWCNT	Conductor	Static-dissipative	Static-dissipative	Conductor
	20 Hz	0.9 Hz	0.1 Hz	300 Hz
	−10^6^	−10^5^	−10^3^	−10^7^
	Metamaterial	Metamaterial	Metamaterial	Metamaterial

^1^ Data from Avila-Orta et al. [22].

## Supplementary Materials

The following are available online at www.mdpi.com/1996-1944/9/11/923/s1.
