# Supplementary material for: Metamaterial Behavior of Polymer Nanocomposites Based on Polypropylene/Multi-Walled Carbon Nanotubes Fabricated by Means of Ultrasound-Assisted Extrusion
Source: Materials (Basel). 2016 Nov 14;9(11):923. doi: 10.3390/ma9110923 (PMC5457228; doi:10.3390/ma9110923)
Supplement: Supplementary file 1 [file materials-09-00923-s001.pdf]

Supplementary Materials: Metamaterial Behavior of Polymer Nanocomposites Based on Polypropylene/Multi-Walled Carbon Nanotubes Fabricated by Means of Ultrasound-Assisted Extrusion

Juan C. Pérez-Medina, Miguel A. Waldo-Mendoza, Víctor J. Cruz-Delgado, Zoe V. Quiñones-Jurado, Pablo González-Morones, Ronald F. Ziolo, Juan G. Martínez-Colunga, Florentino Soriano-Corral and Carlos A. Avila-Orta

Four different fabrication methods were used to fabricate the polymer nanocomposites: W-U (without ultrasound), F-U (fixed-frequency ultrasound-assist fabrication), V-U (variable-frequency ultrasound-assist fabrication), and PT (pretreatment of MWCNT in a fluidized air-bed with an ultrasound probe).

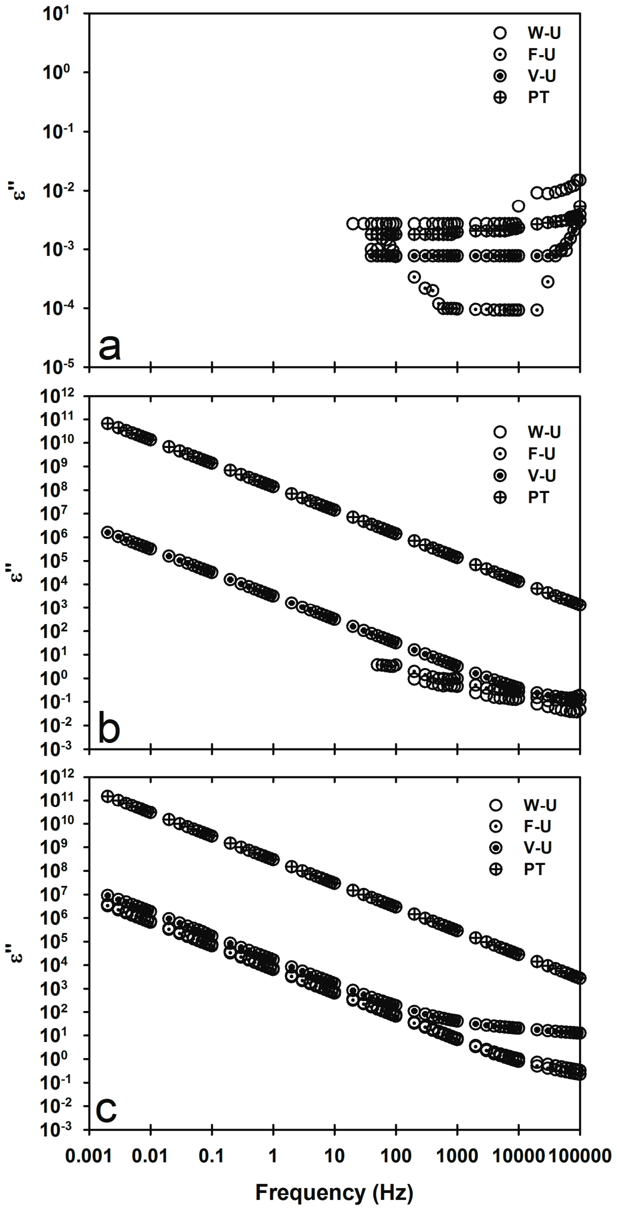

Figure S1. ε″ for (**a**) $iPP_{MFI=2.5}$/MWCNT; (**b**) $iPP_{MFI=34}$/MWCNT; and (**c**) $iPP_{MFI=1200}$/MWCNT fabricated using different ultrasound-assisted extrusion methods.

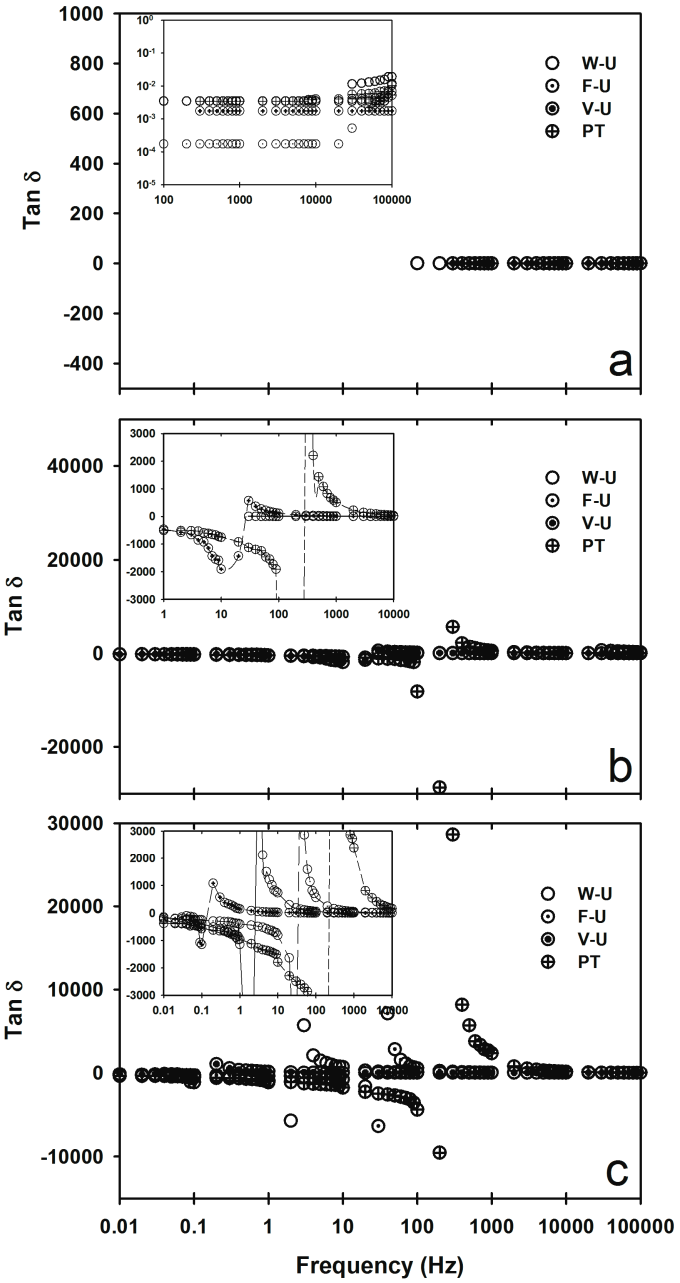

Figure S2. Tan δ for (**a**) $iPP_{MFI=2.5}$/MWCNT; (**b**) $iPP_{MFI=34}$/MWCNT; and (**c**) $iPP_{MFI=1200}$/MWCNT fabricated using different ultrasound-assisted extrusion methods.

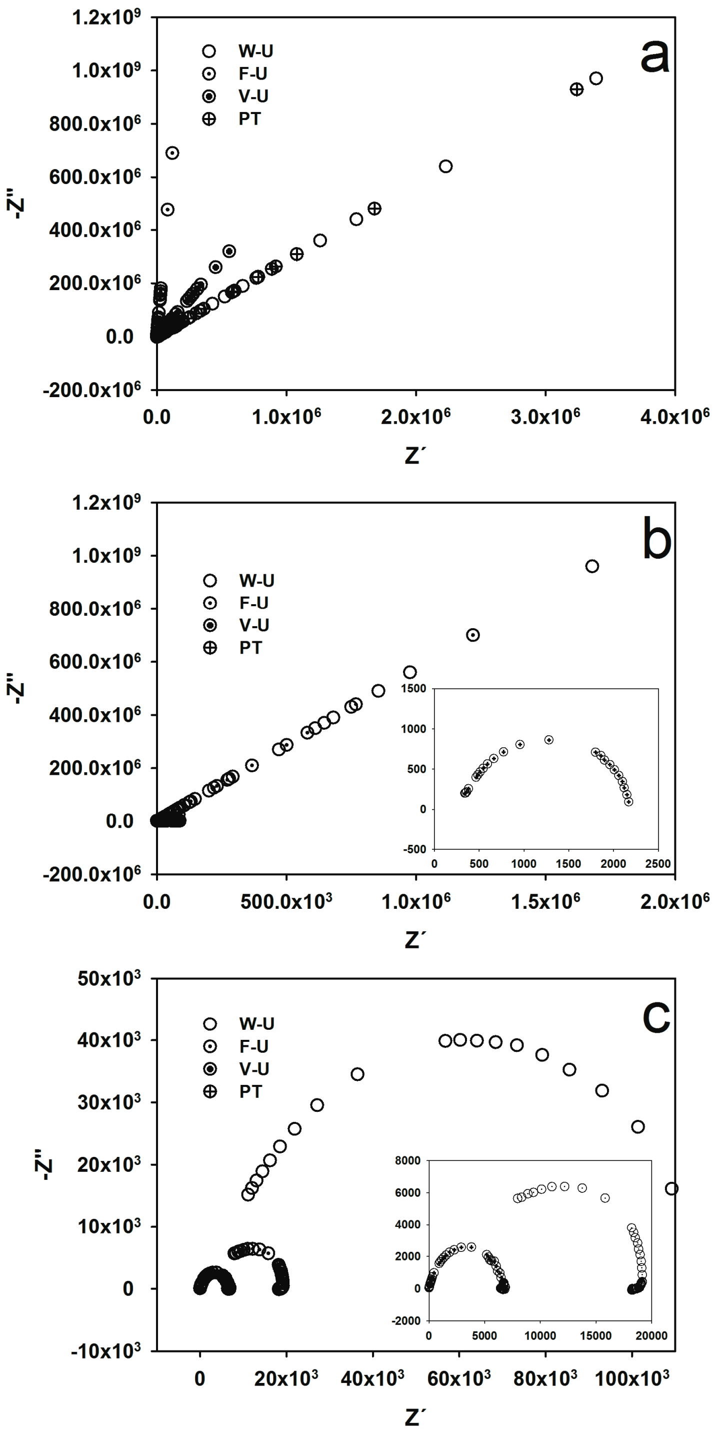

Figure S3. Nyquist plots for (**a**) $iPP_{MFI=2.5}$/MWCNT; (**b**) $iPP_{MFI=34}$/MWCNT; and (**c**) $iPP_{MFI=1200}$/MWCNT fabricated using different ultrasound-assisted extrusion methods.

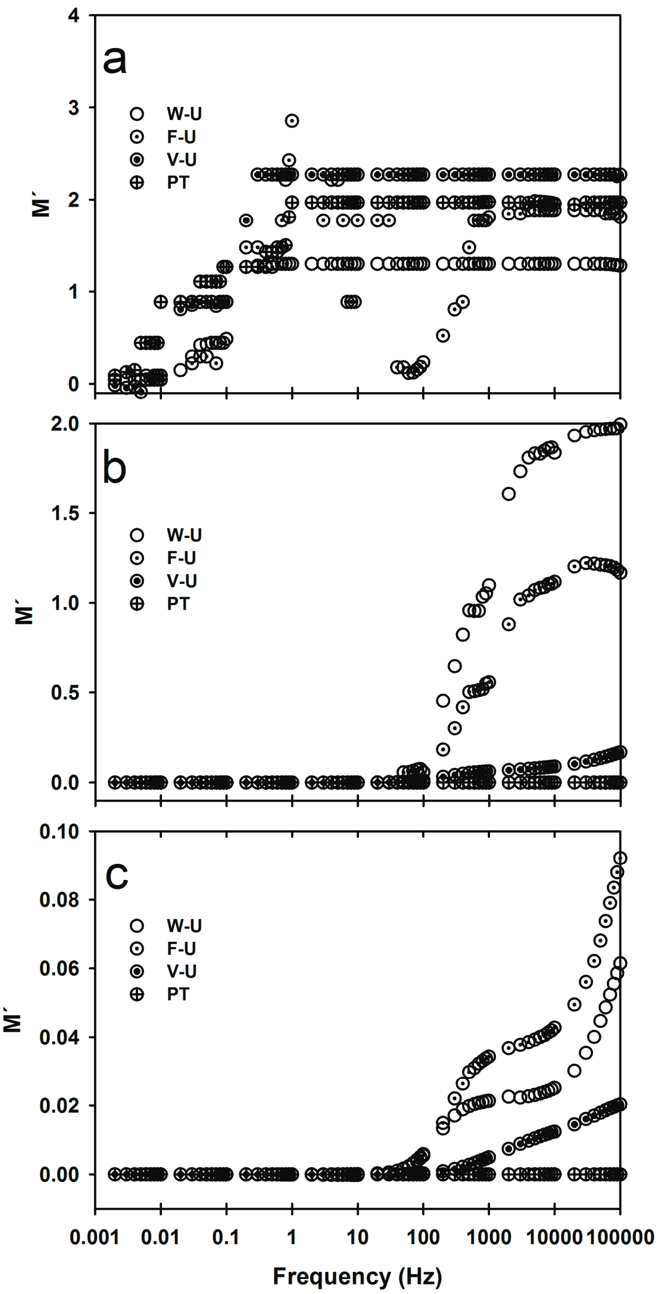

Figure S4. M′ for (**a**) $iPP_{MFI=2.5}$/MWCNT; (**b**) $iPP_{MFI=34}$/MWCNT; and (**c**) $iPP_{MFI=1200}$/MWCNT fabricated using different ultrasound-assisted extrusion methods.

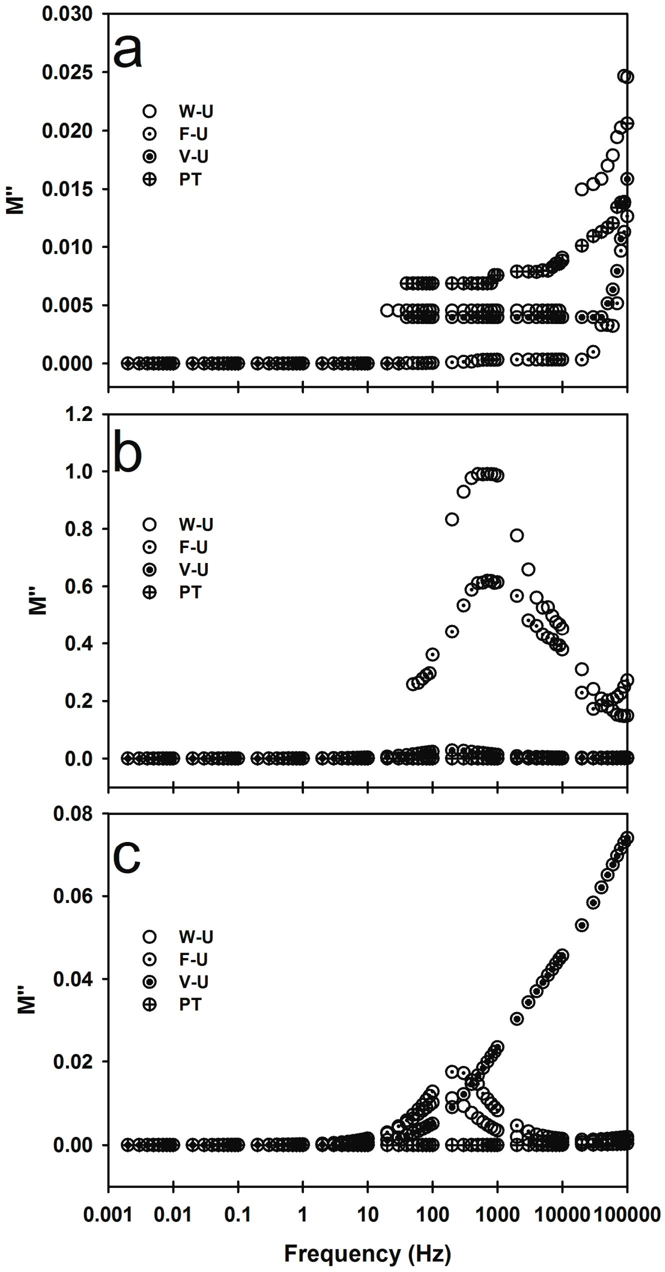

Figure S5. M″ for (**a**) $iPP_{MFI=2.5}$/MWCNT; (**b**) $iPP_{MFI=34}$/MWCNT; and (**c**) $iPP_{MFI=1200}$/MWCNT fabricated using different ultrasound-assisted extrusion methods.

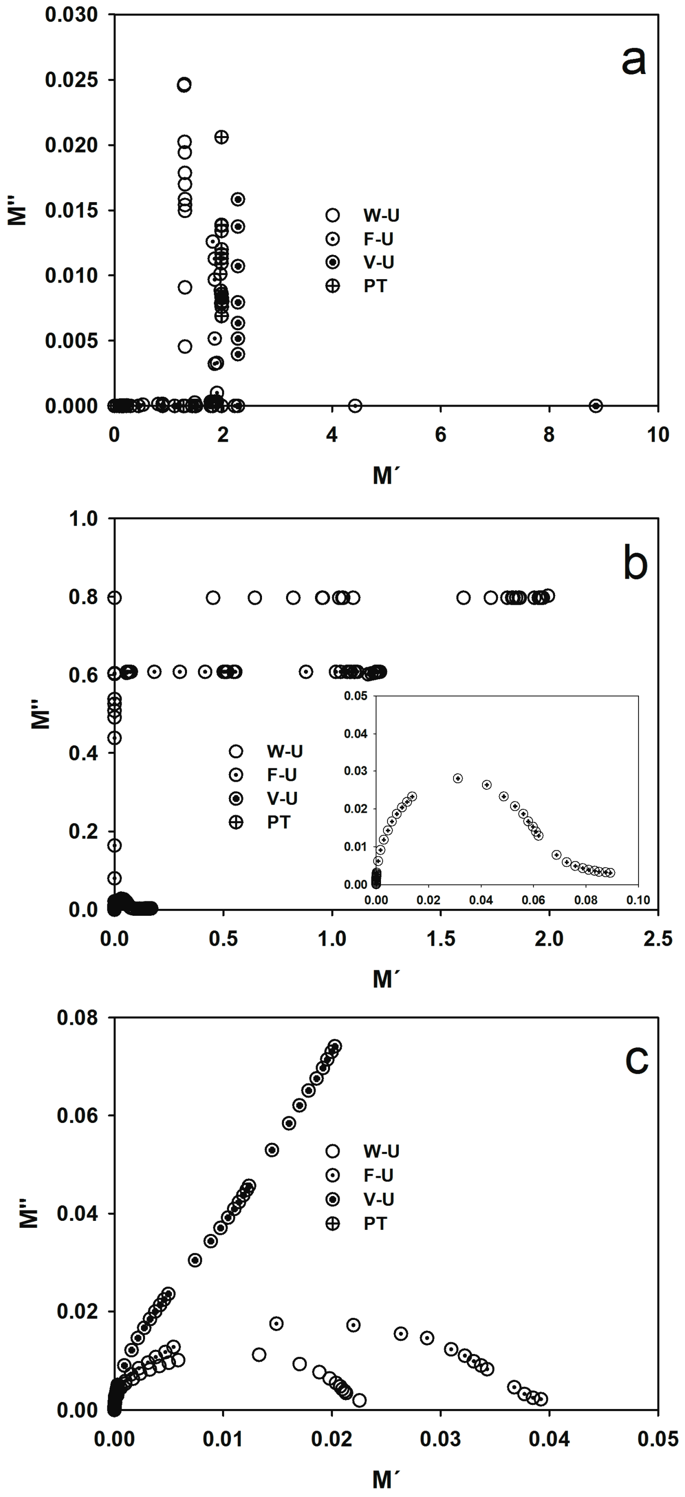

Figure S6. Electrical modulus complex plane (M″ vs. M′) for (**a**) $iPP_{MFI=2.5}$/MWCNT; (**b**) $iPP_{MFI=34}$/MWCNT; and (**c**) $iPP_{MFI=1200}$/MWCNT fabricated using different ultrasound-assisted extrusion methods.

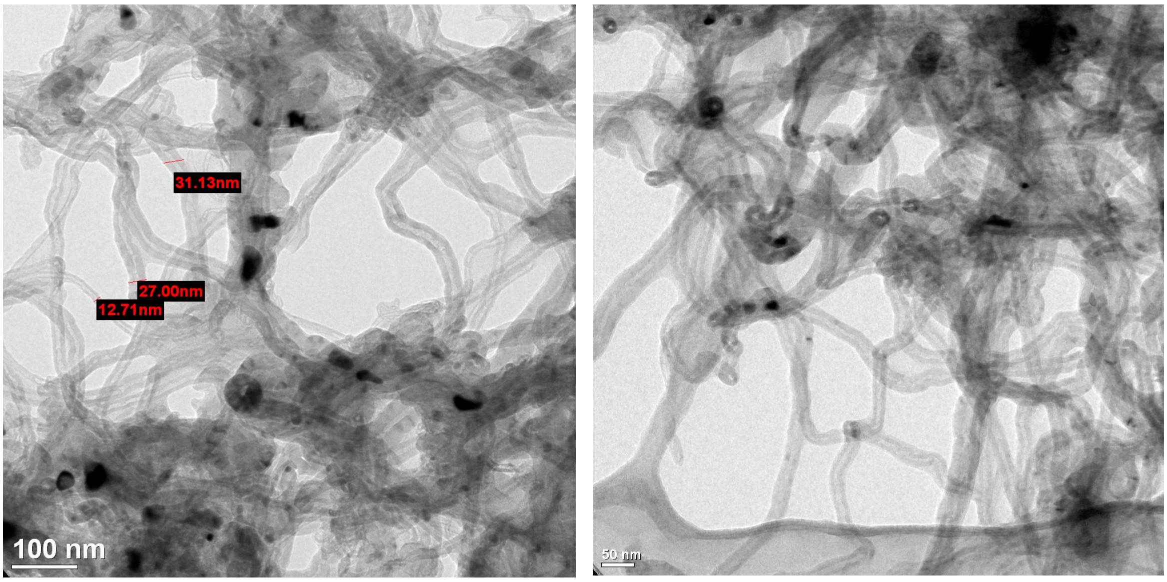

(**a**) (**b**)

Figure S7. TEM micrographs of MWCNTs. (**a**) Lower magnifications; (**b**) Higher magnifications.
